# Innovative Strategies in Oncology: Bacterial Membrane Vesicle-Based Drug Delivery Systems for Cancer Diagnosis and Therapy

**DOI:** 10.3390/pharmaceutics17010058

**Published:** 2025-01-03

**Authors:** Guodong Li, Shuangpeng Pu, Lisiyao You, Yuan Gao, Yuexia Zhong, Huadong Zhao, Dong Fan, Xiyan Lu

**Affiliations:** 1 College of Life Sciences, Northwest University, Xi’an 710069, China; 17836227728@163.com (G.L.);; 2 State Key Laboratory of Holistic Integrative Management of Gastrointestinal Cancers, Biotechnology Center, School of Pharmacy, The Fourth Military Medical University, Xi’an 710032, China; 3 Outpatient Department of the Second Affiliated Hospital of the Fourth Military Medical University, Xi’an 710032, China; 4 Department of General Surgery, Tangdu Hospital, Air Force Medical University, Xi’an 710038, China; zhaolujy@fmmu.edu.cn

**Keywords:** OMV, cancer diagnosis, cancer therapy, drug delivery system

## Abstract

Outer membrane vesicles (OMVs) are double-layered structures of nanoscale lipids released by gram-negative bacteria. They have the same membrane composition and characteristics as primitive cells, which enables them to penetrate cells and tissues efficiently. These OMVs exhibit excellent membrane stability, immunogenicity, safety, and permeability (which makes it easier for them to penetrate into tumour tissue), making them suitable for developing cancer vaccines and drug delivery systems. Recent studies have focused on engineering OMVs to enhance tumour-targeting capabilities, reduce toxicity, and extend circulation time in vivo. This article reviews the latest progress in OMV engineering for tumour treatment and discusses the challenges associated with the use of OMV-based antitumour therapy in clinical practice.

## 1. Introduction

Cancer remains one of the most significant challenges to human health globally, with its incidence and mortality rates continuing to increase [1,2]. Traditional cancer diagnostic and treatment modalities often lack specificity and efficacy, leading to deterioration and progression of the tumours [3]. In recent years, interest in exploring innovative approaches to address these limitations has increased, with a particular focus on nanotechnology-based strategies [4,5]. Among these strategies, bacterial membrane vesicle-based drug delivery systems have emerged as promising options for revolutionising cancer diagnosis and treatment [6].

Bacterial membrane vesicles, particularly outer membrane vesicles (OMVs), are naturally occurring nanosized structures (20–250 nm) produced by gram-negative bacteria during their growth [7,8]. Initially regarded as mere byproducts of bacterial growth, these vesicles have garnered attention for their remarkable properties and potential applications in biomedicine [9]. OMVs possess a lipid bilayer membrane structure that encloses a cargo of proteins, lipids, nucleic acids, and other biomolecules derived from their parent bacteria [10]. This intrinsic composition and structure endow OMVs with several advantageous features that make them attractive candidates for cancer diagnosis and treatment [11]. Compared with other nanoparticles, OMVs have more advantages in the context of drug delivery and cancer therapy. OMVs are naturally produced by bacteria, making them more biocompatible and potentially less toxic than synthetic nanoparticles [7,12]. Their biological origin may also facilitate better interactions with cells and tissues [13]. In addition, OMVs can be engineered to display specific surface proteins or ligands that recognise and bind to certain receptors on cancer cells, allowing customised targeting strategies and precise control over therapeutic delivery [11]. This targeted delivery reduces off-target effects and enhances the efficacy of treatments. Additionally, OMVs can encapsulate a wide range of therapeutic agents, including proteins, nucleic acids, and small-molecule drugs [14,15]. This flexibility allows the development of multifunctional therapeutics tailored to different types of cancer. Most importantly, OMVs can stimulate or modulate immune responses due to their bacterial origin [7]. This property can be harnessed for cancer immunotherapy approaches, potentially increasing the ability of the immune system to recognise and attack cancer cells [16].

Herein, we comprehensively reviewed the biogenesis of OMVs and their applications in cancer therapy and diagnosis. We also summarise modifications to improve the performance of OMVs, shedding light on their potential to revolutionise cancer diagnosis and improve patient outcomes.

## 2. Biogenesis and Formation of Bacterial Outer Membrane Vesicles

**Figure 1:**
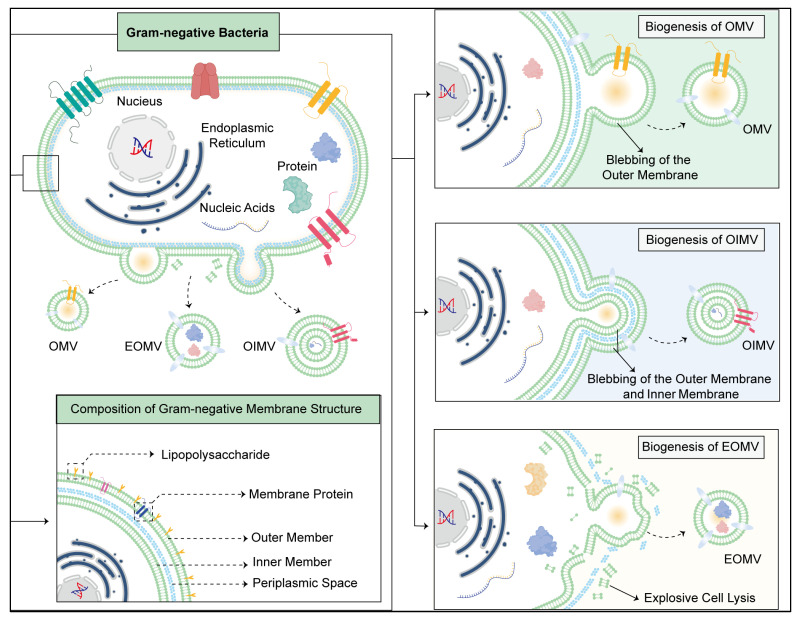
Overview of the process of OMV biogenesis. The biogenesis mechanisms of the three types of bacterial outer membrane vesicles include classic OMVs formed solely by the outer membrane. OMVs are enriched with outer-membrane proteins but no additional cytoplasmic components. OIMVs are formed by the budding of both the inner and outer membranes. EOMVs are formed by explosive cell lysis, which causes cell membrane fragments to recirculate, gather, and randomly wrap cytoplasmic substances to form explosive outer membrane vesicles. Both OIMVs and EOMVs contain envelope material and cytoplasmic components.

The biogenesis of OMVs involves complex cellular processes orchestrated by bacterial envelope machinery [17]. Understanding the intricacies of OMV biogenesis is essential for harnessing these vesicles for various biomedical applications, including cancer treatment [18]. In this overview, we delve into the key steps and regulatory mechanisms underlying OMV biogenesis (Figure 1).

### 2.1. Overview of the Process of OMV Biogenesis

OMVs are derived from the cell membrane vesicles of gram-negative bacteria. Therefore, it is particularly important to clarify the unique structure of bacterial membrane to understand the mechanism of OMV budding and isolation [19]. The outer membrane contains phospholipids and lipopolysaccharides, whereas the periplasmic space contains a layer of peptidoglycan that can protect bacteria from osmotic changes and shear stress [20,21]. In addition, the cytoplasmic membrane consists of the classical phospholipid bilayer (Figure 1).

OMV biogenesis typically begins with the budding of the outer membrane of the bacterial cell [22]. This process can be triggered by various factors, including environmental stresses, such as nutrient limitations, temperature fluctuations, pH changes, and exposure to antimicrobial agents [23]. Additionally, the bacterial growth phase and cell density play significant roles in regulating vesiculation [24]. Under conditions of stress or high cell density, the accumulation of certain molecules in the periplasmic space can induce curvature of the outer membrane, initiating vesicle formation [25]. Once vesiculation is initiated, the bacterial cell mobilises its membrane and periplasmic constituents to assemble nascent OMVs [26]. OMVs include all vesicles that contain the outer membrane produced by gram-negative bacteria and can be divided into two different types according to their formation processes and components [27]. Classical OMVs are derived from membrane materials in living bacteria and are also referred to as B-type MVs. Blebbing of the outer membrane can be a result of cell envelope disturbances [28]. According to their formation patterns, B-type MVs can be subdivided into OMVs and outer-inner MVs (OIMVs) [29] (Figure 1). OMVs are only formed by the outer membrane of bacteria and can be formed by membrane bending, peptidoglycan remodelling, and lipid and protein aggregation [30] (Figure 1). As a result, OMVs are enriched with outer membrane proteins but no additional cytoplasmic components, such as nucleic acids and ATP [31]. In contrast, due to another blebbing mechanism, OIMVs contain not only cell envelope material but also cytoplasmic components [32] (Figure 1). OIMV biogenesis starts with the hydrolysis of peptidoglycan by autolysin [33] (Figure 1). Then, the cytoplasmic membrane protrudes into the periplasm, with the cytoplasmic contents entering the prevesicle. Eventually, the bubbles are squeezed out of the cell surface together with the surrounding outer membrane to form OIMVs [28] (Figure 1). Other types of MVs are called E-type MVs, which are produced via explosive cell lysis [34] (Figure 1). Explosive cell lysis is triggered by genotoxic stress; damage to the DNA of a bacterial chromosome can induce an oxidative stress response and trigger cell death and cleavage, causing cell membrane fragments to recirculate, gather, and randomly wrap cytoplasmic substances to form explosive outer membrane vesicles (EOMVs) [35] (Figure 1). These vesicles play vital roles in the various physiological activities of bacteria. Most studies have shown that OMVs can carry specific receptors that are able to bind nutrients, such as vitamins and amino acids, which are subsequently taken up by bacteria [36,37]. This mechanism not only helps bacteria survive in a competitive environment but also facilitates information exchange and resource sharing among bacteria. Additionally, MVs can be used as excretion tools to expel proteins and lipids that bacteria mistakenly produce due to stress, as well as other metabolic waste [38]. More importantly, MVs can protect bacteria by neutralising membrane-targeting antibiotics and other antimicrobial peptides and complement system factors [39]. Moreover, studies have shown that MVs can facilitate the inter- and intraspecies exchange of DNA. Natalie J. Bitto et al. reported that genomic DNA can be selectively packaged into OMVs during the exponential phase and transferred into host cells [40].

As nascent OMVs mature, the curvature of the outer membrane invaginates further, eventually leading to the constriction and scission of vesicles from the bacterial surface [10]. The precise mechanisms underlying this process remain incompletely understood but likely involve the coordination of various cellular factors, including membrane-remodelling proteins, periplasmic enzymes, and the peptidoglycan layer. Additionally, recent studies have implicated outer membrane proteins, such as BamA and OmpA, in regulating OMV biogenesis and release. Once formed, OMVs are liberated into the extracellular milieu, where they can interact with neighbouring cells or the host environment [41]. Research on the biogenesis mechanism of OMVs has made significant progress. Understanding this mechanism can help us modify OMVs more efficiently and safely. However, further in-depth studies are needed on the selection and release of OMV contents, as well as the key molecules involved in OMV formation, to improve the yield and purity of engineered OMVs.

### 2.2. The Formation of OMVs Is Significantly Influenced by Environmental Factors

The formation of OMVs by gram-negative bacteria is influenced by a multitude of factors, ranging from environmental stimuli to cellular processes. Environmental factors play a significant role in triggering OMV formation as bacteria respond to changes in their surroundings [42,43]. Nutrient availability, temperature fluctuations, pH variations, osmotic stress, and exposure to antimicrobial agents can influence the propensity of bacteria to produce OMVs [44]. For example, nutrient limitation, commonly encountered in natural ecosystems or during infection, can induce stress responses in bacteria, leading to increased vesiculation. Rothfield et al. reported that chloramphenicol exposure and amino acid starvation promote the secretion of these OM blebs in *E. coli* [45]. Moreover, Matthias reported enhanced OMV release upon cysteine depletion [46]. Similarly, shifts in temperature or pH can modulate OMV production. Meta J. Kuehn et al. reported that the lipid A composition in OMVs is directly related to the OMV composition [27]. A neutral pH and high levels of magnesium cations result in stable membrane structures, whereas OMVs produced under acidic conditions are larger and have greater protein density. Additionally, exposure to antimicrobial agents, such as antibiotics or disinfectants, can trigger a defensive response in bacteria, resulting in increased OMV release as a survival strategy. Nonlethal doses of antibiotics can promote the secretion and release of OMVs via activation of the quorum-sensing diffusible signal system. Different living environments have varying impacts on the properties of outer membrane vesicles (OMVs). When using engineered bacteria for in vivo diagnosis and treatment, it is essential to consider the customised environment within the body. Additionally, studying the effects of physical factors such as pH levels and oxygen concentrations on bacterial OMV release can facilitate the clinical translation of probiotics.

### 2.3. Genes Involved in Vesicle Production

OMV biogenesis is subject to intricate regulatory control mediated by various signalling pathways and regulatory networks within bacterial cells. Two-component systems (TCSs), which consist of sensor histidine kinases and response regulators, play pivotal roles in sensing environmental cues and modulating cellular responses, including vesiculation [47,48]. For example, the EnvZ/OmpR system responds to changes in osmolarity, whereas the PhoP/PhoQ system senses magnesium limitation and an acidic pH [49]. Additionally, alternative sigma factors, such as RpoE (σE) and RpoH (σH), coordinate stress responses and envelope integrity, influencing OMV production. Several genes that encode proteins involved in the formation and stabilisation of the outer membrane can also mediate OMV formation, including tolA, tolB, tolC, tolQ, tolR, and pal [50]. At the core of OMV production are genes that encode proteins responsible for maintaining the integrity and stability of the bacterial outer membrane, such as tol-pal genes and bam genes [11]. These genes ensure the proper folding and insertion of outer membrane proteins (OMPs) and contribute to the overall structural integrity of the membrane. Additionally, genes involved in lipopolysaccharide (LPS) biosynthesis, modification, and core region synthesis (such as Lgt and Rfa genes) play crucial roles in determining the composition and properties of the outer membrane, which in turn affects OMV production [51]. Furthermore, genes that encode surface proteins, such as TraT, and outer membrane proteins, such as ompA, are also implicated in OMV production and composition [32]. Overall, understanding the intricate network of genes involved in OMV production provides insights into the biology of gram-negative bacteria and their interactions with the environment. In the future, continued research into the regulatory mechanisms governing OMV biogenesis and the development of genetic manipulation techniques will enhance our understanding of OMV production and may lead to applications in biotechnology, vaccine development, and drug delivery systems.

## 3. Engineered Outer Membrane Vesicles for Cancer Treatment

**Figure 2:**
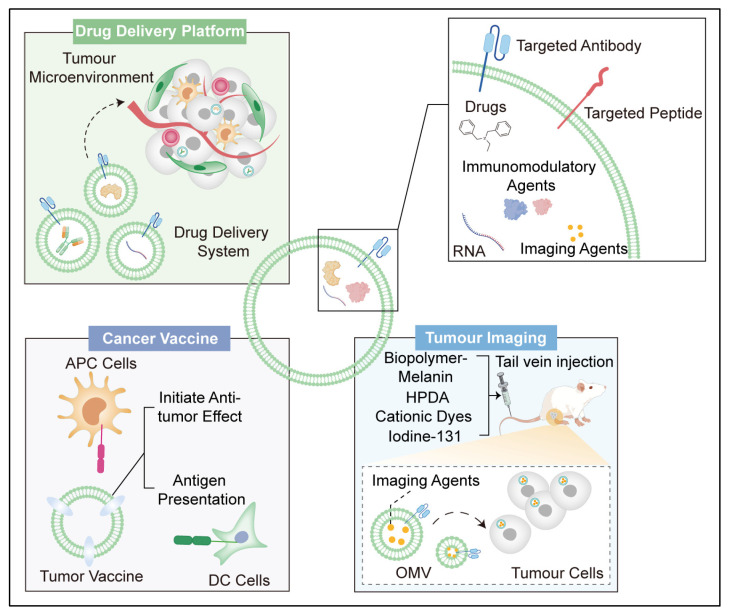
Engineered bacterial outer membrane vesicles (OMVs) for the treatment and diagnosis of tumours: OMVs can encapsulate a variety of tumour therapeutic drugs and diagnostic agents, including RNA, immunotherapeutic drugs, and contrast agents. By serving as carriers for drug delivery, OMVs can deliver treatments such as immunotherapy in a targeted manner, exploiting natural immunogenic advantages to become reliable carriers for tumour vaccines. Additionally, by carrying and intravenously administering contrast agents, OMVs facilitate tumour imaging.

Engineered OMVs have emerged as promising tools for cancer treatment due to their unique properties, including their ability to carry cargo molecules, interact with the immune system, and target specific cells [16]. Here, we review how engineered OMVs are being explored for cancer therapy (Figure 2).

### 3.1. Engineered Outer Membrane Vesicles Act as New Drug Delivery Platforms

OMVs can be engineered to carry various cargo molecules, such as chemotherapeutic drugs, nucleic acids (DNA and RNA), or immunomodulatory agents [52,53] (Figure 2). These cargo molecules can be loaded into OMVs either by passive encapsulation during OMV biogenesis or by active loading techniques. Weng proposed an economic technology to prepare engineered OMVs with pre-miRNA overexpression [54]. They cloned tRNA^lys^ into the pET-31b (+) vector using human genomic DNA as a template. Subsequently, miR-34a, miR-124, and miR-126 precursors were cloned into pET-31b (+)-tRNA to form OMVs overexpressing pre-miRNA stably. They reported that OMVs can inherit tRNA Lys-pre-miRNA from *E. coli* and suppress the growth of breast cancer by significantly inhibiting the expression of the chemokine receptor CXCR4 [54]. Similarly, Vipul Gujrati et al. loaded OMVs with siRNA-TAMRA via an electroporation method to target kinesin spindle protein, which resulted in highly significant tumour growth regression in an animal model [55]. OMVs can also deliver chemotherapy drugs. Kudelaidi et al. gently mixed DOX and OMVs and then incubated them at 37 °C for 4 h to obtain DOX-loaded OMVs. In vitro and in vivo experiments revealed that DOX-OMV not only substantially inhibited tumour growth but also recruited macrophages and elicited suitable immune responses [56]. Zhuang et al. constructed bacteria-plant hybrid vesicles (BPNs) by simply fusing thylakoid membranes with OMVs. These BPNs with photodynamic effects derived from thylakoids can directly lead to the disruption of local tumours under laser irradiation [57]. Su et al. developed a genetically encoded strategy for lysosomal degradation (lysosome-targeting chimaera, LYTAC) via engineered OMVs [58]. The transferrin receptor (TfR) is a promising lysosomal-targeting receptor, and the ClyA protein is one of the most abundant and easily modified proteins on the surfaces of OMVs. They engineered a PD-L1-targeting TfR-LYTAC to ClyA, which achieved the targeted degradation of PD-L1 in lysosomes and rapid regression of tumour tissue. The membrane of OMVs has a negative charge (zeta potential: ~ −13 mV) and can be closely combined with anionic compounds via electrostatic interactions. Liu combined OMVs with cationic dyes (IR780, Cy7, and Cy7.5), which exhibit excellent phototherapy of tumours without any major signs of toxicity [59].

Moreover, a recent study showed that OMVs can effectively encapsulate oncolytic adenoviruses. Ban et al. explored engineered OMVs for autophagy cascade-augmented immunotherapy [60]. They first produced P_2_O-expressing OMVs via plasmid transfection and then extruded OMVs with adenoviruses via a liposome extruder to acquire engineered OMVs, which can lead to excessive H_2_O_2_ accumulation and increased oxidative stress levels to trigger tumour autophagy. Even though OMVs can be engineered for strong tissue and cell targeting in basic experiments, most OMVs are actually taken up by mononuclear phagocyte system organs such as the liver and spleen. This significantly reduces their circulation time in the body, leading to a decrease in the effective concentration of the drug. Increasing the circulation time of OMVs in the body and further enhancing their targeted concentration in the desired tissues are crucial steps for the clinical application of OMVs.

### 3.2. Engineered Outer Membrane Vesicles Act as Cancer Vaccines

Engineered outer membrane vesicles (OMVs) hold great potential as cancer vaccines because of their unique ability to stimulate the immune system and induce antitumour immune responses [61,62] (Figure 2).

**Figure 3:**
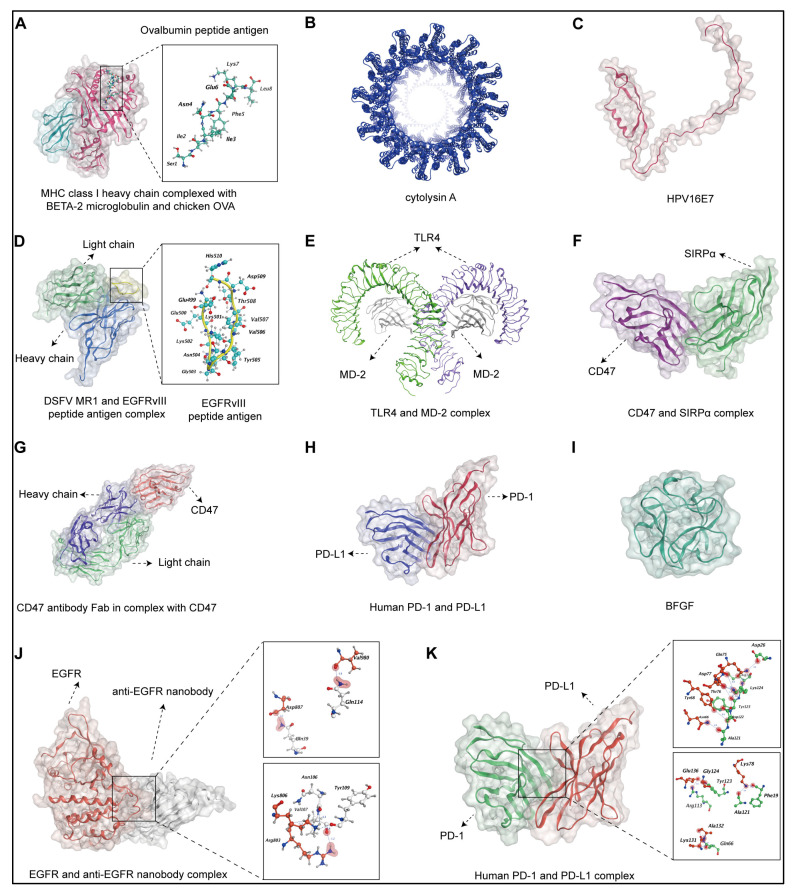
The structures of proteins shown by the molecular operating environment (MOE). (**A**) MHC class I heavy chain (red) complexed with BETA-2 (blue) macroglobulin and chicken ovalbumin (green). The top right picture shows the structure and amino acid compounds. (**B**) The protein structure of cytolysin A. (**C**) The predicted protein structure of HPV16E7. (**D**) DSFV MR1 (the heavy chain and light chain are marked blue and green, respectively) and the EGFRvIII peptide antigen (yellow) complex. The EGFRvIII peptide antigen structure and amino acid compounds are shown at the top right. (**E**) Structures of TLR4 (violet and green) and the MD-2 complex (grey). (**F**) The protein structures of the CD47 (violet) and SIRPα (green) complexes. (**G**) The complex protein structure of the CD47 antibody Fab (the heavy chain is marked in violet, and the light chain is marked in green) in complex with CD47 (red). (**H**) The human protein structure complex of PD-1 (red) and PD-L1 (violet). (**I**) The protein structure of BFGF. (**J**) The molecular docking results revealed the interaction between EGFR and the EGFR nanobody. (**K**) Interaction analysis via the MOE revealed the interaction between human PD-1 and PD-L1.

Antigen Presentation: OMVs can be loaded with tumour-specific antigens, either by engineering the parent bacteria to express these antigens or by directly incorporating them into the vesicles during biogenesis (Figure 2). These antigens can be derived from tumour cells, tumour-associated antigens, or neoantigens specific to individual patients [63]. Schetters et al. reported that the stable expression of ovalbumin (OVA) on the surfaces of OMVs can effectively cross-commit into OT1 cells through BMDCs and spleen CD11c^+^ DCs via MyD88 and Toll-like receptor signalling, which can induce OVA-specific tumour clearance [64]. Here, we show the structure of the protein complex, which is an MHC class Ⅰ heavy chain complexed with BETA-2 microglobulin and OVA (Figure 3A). OVA combines with the MHC class I heavy chain, and the amino acids sequence of OVA is shown in Table S1. Similarly, Zhao et al. designed a novel antigen delivery platform that can modify *E. coli*-released OMVs to carry specific tumour antigens fused with the protein cytolysin A [65]. The structure of the protein cytolysin A is shown in Figure 3B. These OMVs can cross the epithelium into the lamina propria and stimulate dendritic cell maturation to generate long-term cancer-killing immunity. Wang et al. synthesised an engineered *E. coli* strain that can release HPV16E7 peptide-expressing OMVs [66]. These bacterial vesicles can promote tumour-specific CD4- and CD8-positive cytotoxic T lymphocyte responses in TC-1 tumour-bearing mice and might be applied in clinical practice. The predicted peptide structure of HPV16E7 contains 98 amino acids, as shown in Figure 3C. Moreover, Grandi et al. fused three copies of EGFRvIIIpep DNA to the 3′ end of the Neisseria meningitidis fHbp gene to produce EGFRvIII tri-peptide-attached OMVs, which can protect mice from tumour challenges [67]. We identified the complex protein structure of DSFV MR1 and the EGFRvIII tri-peptide (Figure 3D), and the EGFRvIII peptide antigen consists of 12 amino acids (Table S1). Yue et al. modified *E. coli* to express a specific tumour antigen fused with cytolysin A on the surfaces of OMVs under the control of a promoter induced by the monosaccharide arabinose, which exhibited a strong antitumour effect [68]. Furthermore, they also created a novel versatile antigen display platform based on OMVs for tumour vaccination via plug-and-display technology. They coexpressed SpC and SnC with ClyA on the OMV surface, while SpT- or SnT-labelled tumour antigens can be displayed rapidly on OMVs by binding to ClyA catchers through isopeptide bonds between the tag and catchers. This technology provides a rapid, economic, and universal approach to presenting various tumour antigens to DCs.

Immunostimulatory Molecules: Engineered OMVs can be modified to express immunostimulatory molecules, such as Toll-like receptor (TLR) agonists, cytokines, or costimulatory molecules [69,70]. These molecules enhance the activation and maturation of antigen-presenting cells (APCs) and promote the generation of robust antitumour immune responses (Figure 2). Natural OMVs derived from *E. coli* can elicit immune activation and initiate antitumour effects by facilitating the recognition and destruction of malignant cells. Firth et al. demonstrated that ΔlpxM OMVs can stimulate PBMCs and Vγ9Vδ2 T cells [71]. Additionally, they reported that activated Vγ9Vδ2 T cells had a robust cytolytic effect on breast cancer cells and leukaemia cells after OMV-mediated expansion. Parseh et al. activated natural killer cells and transformed M2 macrophages into M1 macrophages using tumour-targeting OMVs via TLR4 and other TLR-activating components on the surface of OMVs [72]. The structure of the complex containing TLR4 and MD-2 is shown in Figure 3E. However, studies have modified OMVs to improve their immune stimulation effects. Blocking CD47-SIRPα signalling pathway-mediated tumour cell immune escape could improve antitumour effects. Liang et al. developed SIRPα-Fc-assisted OMVs that can trigger TAM-mediated phagocytosis to exert vaccine-enhanced antitumour activity [73]. Similarly, Feng et al. reported that CD47 nanobody-modified OMVs can effectively activate TAM phagocytosis of tumour cells via multiple pathways, including the induction of M1 polarisation and blockade of the “do not eat me” signal [74]. The complex peptide structures of CD47 and SIRPα are shown in Figure 3G. The structure of the CD47 antibody in complex with CD47 is shown in Figure 3F. Additionally, Li et al. reported that an ectodomain of programmed death 1 (PD-1)-containing OMVs protects T cells from the PD-1/PD-L1 immune inhibitory axis, which can comprehensively regulate the tumour immune microenvironment and markedly increase antitumour efficacy [75]. Furthermore, we show the protein structure, which contains PD-1 and PD-L1, in Figure 3H. Basic fibroblast growth factor (BFGF) is one of the key factors in mediating tumour growth processes, including tumour angiogenesis, antiapoptotic effects, and immune escape. Xie et al. developed a novel antitumour system based on BFGF-loaded OMVs that can induce persistent autoantibodies in mice to antagonise BFGF functions and therefore block tumour angiogenesis [76]. They successfully demonstrated that these BFGF antibodies induced by OMVs can reverse the immunosuppressive microenvironment and increase the lymphocyte (CTL) reaction. The protein structure of BFGF is shown in Figure 3I. Interestingly, Cheng et al. reported that tumour antigens captured by OMVs generated from photosynthetic bacteria (PSB) can efficiently present tumour antigens in the tumour periphery and TDLNs and increase the infiltration of CTLs into a tumour under a near-infrared (NIR) laser [63]. These exciting phenomena indicate that OMVs derived from PSB carry some photosensitive compounds that can trigger immune reactions via NIR irradiation. Engineered OMVs exhibit substantial potential in the realm of cancer immunotherapy, heralding a promising avenue for the development of personalised and highly efficacious vaccines. The unique appeal of OMVs lies in their dual functionality, they can be meticulously engineered to display tumour-specific antigens while simultaneously serving as potent adjuvants, thereby amplifying the immune response due to their intrinsic immunogenicity. This synergistic combination holds the promise of generating vaccines capable of eliciting robust and targeted immune responses, potentially offering innovative therapeutic options for cancers that are currently challenging to treat. However, whether all bacterial-derived OMVs possess adjuvant effects, the differences in immune responses elicited by OMVs from different bacterial sources, and whether OMVs from various bacterial origins can be applied to different immunological scenarios (such as antiviral immunity and antibacterial immunity) are critical questions that require urgent investigation in the future.

### 3.3. Engineered Outer Membrane Vesicles for Tumour Imaging

Due to their unique properties, engineered OMVs have emerged as promising tools for tumour imaging. By engineering OMVs with specific targeting ligands, such as peptides or antibodies, researchers can precisely target tumours [16]. Additionally, OMVs can be loaded with imaging agents (Figure 2), such as fluorescent dyes or nanoparticles, allowing the real-time visualisation of tumours by imaging techniques such as fluorescence microscopy or positron emission tomography (PET) [77]. This approach holds great potential for improving the early detection and monitoring of tumours in cancer patients (Table 1). Gujrati et al. reported a new tumour imaging system based on OMVs that encapsulate the biopolymer melanin (OMV^Mel^) via tyrosinase transgene bacteria [77] (Figure 2). They reported that OMV^Mel^ absorbed in tumour tissues results in strong optoacoustic signals from the absorbed laser energy, which is appropriate for imaging applications. Similarly, Wang et al. reported on OMV-cancer cell membrane hybrid vehicles and coated these materials onto hollow polydopamine (HPDA) NPs (Figure 2), which can also be applied for tumour imaging [78]. Additionally, we reviewed cationic dye-loaded OMVs generated by electrostatic interactions and reported that the efficient accumulation of OMVs in tumours can not only induce tumour apoptosis but also enable sensitive tumour detection via optoacoustic technology. Liu et al. demonstrated that cationic dyes, such as IR780, Cy7, and Cy7.5, form stable complexes with negatively charged bacterial outer membrane vesicles (OMVs). This interaction enhances the dyes’ in vivo circulation and optoacoustic properties, making them more effective for biomedical applications [79]. Additionally, ^131^I-labelled OMVs taken up by tumour tissues were proven to have high radio labelling efficiency and stability and could be detected by SPECT/CT imaging [80]. Current research predominantly focuses on enhancing the tumour-targeting capability of OMVs to enable their use in tumour imaging. However, the issue of tumour-specific imaging remains inadequately addressed. If OMVs could be developed to carry molecules that respond specifically to tumour-related stimuli, such as binding to certain substances within the tumour and generating signals like inflammation changes, pH alterations, or changes in emitted light, the effectiveness of OMVs in tumour imaging could be significantly improved.

## 4. Modification of Outer Membrane Vesicles for Better Performance

OMVs have garnered significant attention in biomedical research because of their unique properties and potential applications [76,81]. These nanosized vesicles, which are naturally released by gram-negative bacteria, possess a lipid bilayer structure that contains various proteins and lipids derived from the bacterial outer membrane. Researchers have increasingly focused on harnessing the versatility of OMVs for biomedical purposes, including drug delivery, vaccine development, and diagnostic imaging. In particular, the modification of OMVs has emerged as a promising strategy to increase their performance for specific applications [61].

### 4.1. Modification of Outer Membrane Vesicles for Long Circulation

**Figure 4:**
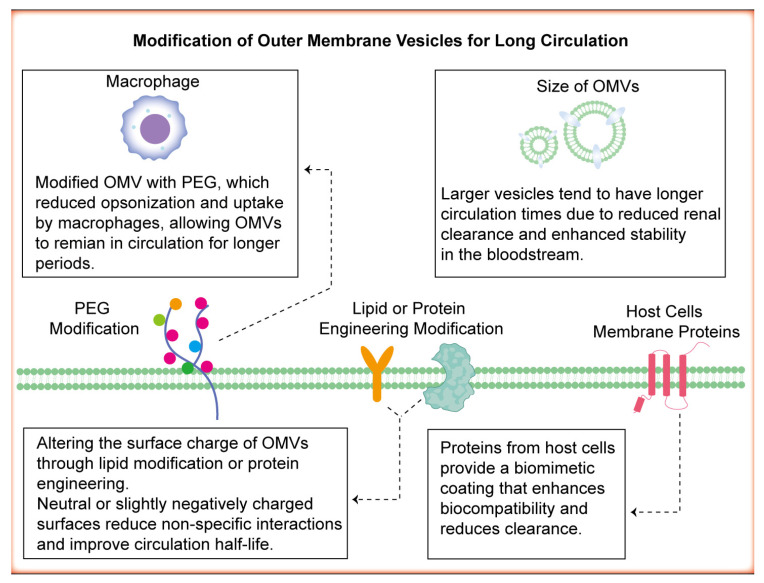
Modified OMVs for extended circulation time in the bloodstream. PEGylation can effectively reduce macrophage phagocytosis of OMVs. Controlling the size of OMVs can influence their circulation kinetics. Modifications with host-derived membrane proteins can effectively prevent immune recognition and clearance. Furthermore, lipid and protein engineering modifications influence the surface charge of OMVs.

Modification of OMVs for prolonged circulation in the bloodstream is crucial to enhance their effectiveness in cancer therapy and diagnosis, including drug delivery and imaging [82]. A prolonged circulation time increases the likelihood of OMVs reaching their target site, thereby increasing their therapeutic or diagnostic efficacy while minimising systemic clearance. Coating OMVs with polyethylene glycol (PEG) molecules can shield them from recognition and clearance by the immune system, extending their circulation time [83] (Figure 4). PEGylation reduces opsonisation and uptake by macrophages, allowing OMVs to remain in circulation for longer periods (Figure 4). Shen et al. integrated the tyrosine-rich protein statherin onto the surfaces of OMVs and then modified STATH–OMVs with PEG, which resulted in longer circulation times and increased tumour accumulation in OMVs [37]. DSPE-PEG-RGD-modified OMVs also exhibited improved blood circulation time in melanoma mice [84]. Alternatively, controlling the size of OMVs can influence their circulation kinetics [85] (Figure 4). Larger vesicles tend to have longer circulation times because of reduced renal clearance and enhanced stability in the bloodstream. However, size should be optimised to avoid rapid clearance by the RES while ensuring efficient delivery to target tissues [86]. Furthermore, enhancing the surfaces of OMVs with cell membrane proteins from host cells can camouflage them, preventing recognition by the immune system and prolonging circulation [87] (Figure 4). These proteins provide a biomimetic coating that enhances biocompatibility and reduces clearance. Finally, lipid bilayers need to come into contact and mix for vesicle fusion to take place. Altering the surface charge of OMVs through lipid modification or protein engineering can influence their interaction with plasma proteins and immune cells [88] (Figure 4). Neutral or slightly negatively charged surfaces reduce nonspecific interactions and improve the circulation half-life (Figure 4). To sum up, OMVs can be modified for extended circulation time in the bloodstream. PEGylation can effectively reduce macrophage phagocytosis of OMVs. Controlling the size of OMVs can influence their circulation kinetics. Modifications with host-derived membrane proteins can effectively prevent immune recognition and clearance. Furthermore, lipid and protein engineering modifications influence the surface charge of OMVs. Extending the circulation time of OMVs holds clinical significance for increasing their concentration in target organs. However, if the specific mechanisms of cellular uptake of OMVs can be elucidated and OMVs can be engineered to achieve cell-specific uptake, it would be possible to enhance the concentration of OMVs in target organs without affecting their pharmacokinetics.

### 4.2. Modification of Outer Membrane Vesicles for Improved Special Targeting

**Figure 5:**
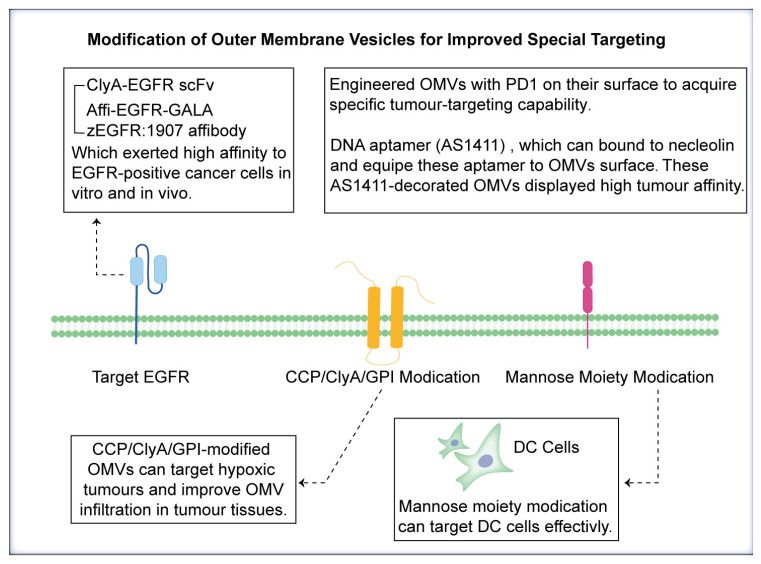
Modification of outer membrane vesicles to improve special targeting. The scFv-targeting EGFR, combined with ClyA modification, exhibits high efficiency in EGFR targeting. PD-1 and AS1411 modifications on the surfaces of engineered OMVs can specifically target tumour cells. Modifications such as ClyA/OmpA can enhance infiltration in tumour tissues. Additionally, modification with the mannose moiety can specifically target DC cells.

Cancer therapy continues to evolve, with a growing emphasis on targeted approaches to improve treatment outcomes. OMVs have emerged as promising candidates for drug delivery because of their natural ability to encapsulate biomolecules and their potential for precise targeting [54] (Table 2). Rezaei et al. introduced ClyA-EGFR scFv-expressing OMVs, which exhibited high affinity for EGFR-positive cancer cells both in vitro and in vivo [89] (Figure 4). Similarly, Sepahdar et al. fused the affi-EGFR-GALA structure to the C-terminus of ClyA as an anchor protein to increase the degree of OMV targeting [90]. The GALA peptide, a pH-responsive amphipathic peptide, enables OMVs to escape the endosome, whereas the zEGFR:1907 affibody enables OMVs to anchor to EGFR [91]. Here, we obtained the crystal structures of EGFR and the anti-EGFR nanobody, and molecular docking revealed strong interactions between them. EGFR interacts with anti-EGFR nanobodies via hydrogen bonding (Figure 3J), and the amino acids involved in such interactions are shown in Table S1. The interaction results further revealed the reliability of targeting EGFR. In line with these studies, Li et al. and Cui et al. engineered OMVs with programmed death receptor 1 (PD-1) on their surfaces to acquire specific tumour-targeting capabilities [75,92]. Here, we obtained the crystal complex structure of human PD-1 and PD-L1 and analysed the interactions between PD-L1 and its receptor by the MOE, and the methods are shown in the Supplementary Materials. The interaction results revealed that PD-1 interacts with PD-L1 via hydrogen bonding (Figure 3K), and the amino acids involved in such interactions are shown in Table S1. The interaction results revealed the effectiveness of targeting PD-L1 via PD-1 modification on MOVs, which specifically bind to tumours. Nucleolin is a specific protein selectively expressed on tumour cell membranes [93]. Chen et al. designed a DNA aptamer (AS1411) that can bind to nucleolin and equipped these aptamers to the surfaces of OMVs via hydrophobic interactions between cholesterol and the lipid membrane [94]. These AS1411-containing OMVs displayed high tumour affinity. Passive targeting strategies are also an important way to improve the tumour targeting ability of OMVs. The PEG modification described earlier is an important advantage for improving not only the circulation time of OMVs but also their aggregation in tumour tissue [95]. Furthermore, Wang et al. introduced poly(L-arginine) cell-penetrating peptide (CPP)-modified OMVs to improve OMV infiltration in tumour tissues [96]. The accumulation of extracellular matrix (ECM) macromolecules, such as hyaluronic acid, is one of the most important features of solid tumours, which hinders the infiltration of immune cells and drug delivery [97]. Shindu C Thomas reported that cytolysin A (ClyA)-hyaluronidase (Hy)-overexpressing OMVs can target hypoxic tumours and remodel the tumour stroma [98,99]. In addition to the ClyA fusion protein, modification of OMVs with glycosylphosphatidylinositol (GPI)-anchored proteins is also a promising approach. Marianne et al. reported that the lipid part of the GPI anchor is inserted into cell membranes, and that two completely different GPI proteins can be displayed on the same surfaces of OMVs [100,101] (Figure 5). In contrast, Li et al. first bioengineered OMVs conjugated with TiO_2_-coated Fe_3_O_4_ NPs to construct magnetically propelled macrophage-based microrobots, which made magnetic-induced aggregation in cancer possible [102]. In addition to cancer targeting, immune cell targeting is also an effective strategy for cancer therapy. Nie et al. constructed a single-shot prophylactic tumour vaccine enabled by an injectable biomembrane hydrogel based on mannose-decorated OMVs [18]. The mannose moiety, which enables active targeting, facilitated active targeting to dendritic cells (DCs) and effective DC maturation [103] (Figure 5). However, Zhao reported that different charges on the surfaces of OMVs result in different DC uptake efficiencies [104]. Compared with negatively charged and neutral OMVs, positively charged OMVs have elevated DC uptake but weakened antitumour immunity. As a result, the use of electrostatic forces to display positively charged tumour antigens on negatively charged OMVs might be a novel strategy to improve DC targeting [105]. The modification of OMVs for enhanced targeting represents a promising avenue for advancing cancer therapy. By incorporating targeting ligands, engineering tumour-homing proteins, and leveraging stimuli-responsive or biomimetic approaches, researchers can tailor OMVs to precisely deliver therapeutic payloads to cancer cells [106]. These advancements hold great potential to revolutionise cancer treatment paradigms, offering new opportunities for personalised and effective therapies and diagnoses.

### 4.3. Modification of Outer Membrane Vesicles for Improved Biosecurity

In the realm of biotechnology, OMVs hold promise for diverse applications, from targeted drug delivery to vaccine development [107]. However, concerns regarding biosecurity risks associated with their use have prompted researchers to explore modifications that enhance containment and mitigate potential threats. Selective targeting strategies can be employed to ensure the precise delivery of therapeutic payloads while minimising off-target effects, thus bolstering biosecurity. However, completely reducing the immunogenicity and toxicity of OMVs from the source is fundamental [7]. The toxicity of LPS to OMVs remains a major concern, especially when it is applied in the human body [108]. The MsbB gene plays a pivotal role in the biosynthesis of LPS in *E. coli* [51]. Most studies select the ∆msbB/∆pagP mutant of nonpathogenic *E. coli* as the OMV producer, where the lipid component of lipopolysaccharide is inactivated [109]. Yong Song Gho demonstrated that OMVs derived from ∆msbB *E. coli*, which lack the lipid A acyltransferase (msbB), do not activate the TLR4 receptor. Consequently, these OMVs do not induce the production of interleukin-8 (IL-8), thereby mitigating the toxicity associated with inflammatory stress [110]. The phoP/phoQ locus, which consists of a membrane-associated sensor kinase (phoQ) and a cytoplasmic transcriptional regulator (phoP), modulates important virulence functions, including resistance to endogenous antimicrobial peptides and the bacterial survival rate in macrophages [111]. PhoP/PhoQ-depleted *Salmonella typhimurium* has been shown to play a promising role in vaccine application and as a vehicle for plasmid delivery. Designing auxotroph strains is another effective strategy to reduce toxicity. Auxotrophic strains can reportedly absorb necessary amino acids from the tumour environment but do not persist in normal tissues [112]. Additionally, the disruption of aroA and rfaH can generate an LPS-deficient strain based on these auxotroph strains [113]. An alternative strategy involves modifying the surface of OMVs with synthetic coatings, such as chitosan, PEG, polydopamine, and other lipids [114]. Above all, regulatory oversight and risk assessment frameworks play crucial roles in ensuring the safe and responsible use of engineered OMVs, guiding the development of comprehensive strategies to mitigate potential risks.

## 5. Perspectives

Recently, OMVs have attracted attention as new engineering platforms for cancer diagnosis and therapy [115]. In cancer diagnosis, OMVs offer several advantages. They can be engineered to display tumour-specific antigens or biomarkers on their surfaces, facilitating their recognition and binding to cancer cells [16]. This targeted approach enhances the specificity and sensitivity of diagnostic assays, which improves the accuracy of cancer detection at an early stage. Moreover, OMVs can encapsulate imaging agents, such as fluorescent dyes or contrast agents [77]. Such OMVs can be utilised in noninvasive imaging modalities, such as fluorescence microscopy or magnetic resonance imaging (MRI), to visualise tumours with high resolution. Furthermore, OMVs hold great potential in cancer treatment. They can serve as carriers of various therapeutic payloads, including chemotherapeutic drugs, nucleic acids, or immunomodulatory agents [6]. Encapsulating these agents within OMVs improves their stability and facilitates their controlled release, which prolongs the circulation time and improves bioavailability. Additionally, OMVs can be engineered to target specific receptors or pathways that are overexpressed in cancer cells, thereby minimising systemic toxicity and maximising therapeutic efficacy [60].

Compared to chemically synthesised polymeric particles, OMVs exhibit superior biocompatibility [11]. Additionally, modifications to OMVs have been shown to further reduce cytotoxicity, enhancing their safety profile. This makes OMVs particularly advantageous for biomedical applications, including drug delivery and therapeutic interventions, where minimising adverse effects is crucial. Their natural composition and ability to be engineered for specific functions underscore their potential as a versatile and effective platform in nanomedicine. Exosomes, liposomes, and OMVs are promising vectors for drug delivery in tumour treatment. Among these, OMVs stand out due to their inherent immunogenicity, which can trigger a robust innate immune response [13]. This characteristic makes OMVs not only effective drug carriers but also natural adjuvants for tumour vaccines, enhancing and sensitising the immune response against cancer cells. Additionally, OMVs’ ability to carry a diverse range of bioactive molecules further underscores their potential in targeted cancer therapy and immunotherapy.

Despite their immense potential, several challenges need to be addressed to realise the full clinical utility of OMVs in cancer diagnosis and treatment. One challenge is the heterogeneity of tumours, which may require the development of personalised OMV-based therapies tailored to individual patients [116]. Another challenge is the scalability of OMV production; current methods rely on bacterial cultures, which may limit their widespread use in clinical settings [117]. Despite these exciting perspectives, several challenges need to be critically examined:

Safety and Immunogenicity Control: While OMVs are naturally immunogenic, this very property can pose a safety risk. The lipopolysaccharides (LPS) in OMVs can lead to excessive immune activation, potentially causing harmful inflammatory responses or even toxic shock. Engineering OMVs to retain their immune-stimulating properties while avoiding hyperinflammatory reactions is a delicate balance that requires further research.

Antigen Delivery and Specificity: Loading OMVs with tumour-specific antigens raises questions about the efficiency and consistency of antigen delivery. Not all OMVs may be equally capable of incorporating the desired antigens, leading to variability in vaccine efficacy. There is also the challenge of ensuring that the antigens presented are relevant to a patient’s specific tumour profile, as cancers can be highly heterogeneous. Developing strategies for precise antigen selection and loading is crucial for the success of OMV-based vaccines.

Immune Tolerance and the Tumour Microenvironment: One of the significant obstacles in cancer immunotherapy is overcoming the immunosuppressive nature of the tumour microenvironment. Tumours often develop mechanisms to evade immune detection, such as inducing tolerance in immune cells or secreting immunosuppressive molecules. It remains to be seen whether OMV-based vaccines can effectively overcome these barriers and trigger a potent immune response in the context of an immunosuppressive tumour microenvironment. Understanding how OMVs interact with immune cells and whether they can bypass these suppressive signals is critical.

Regulatory and Manufacturing Hurdles: Producing OMVs at a clinical grade requires stringent quality control, particularly when engineered for therapeutic purposes. The manufacturing process must ensure that OMVs are free from contaminants, such as residual bacterial components that could provoke undesirable side effects. Moreover, each batch must maintain consistency in terms of antigen loading and vesicle properties, which may be technically challenging. Regulatory approval for such biologics will demand comprehensive safety and efficacy data, which could delay their clinical adoption.

Long-term Efficacy and Memory Response: While OMVs can stimulate strong immune responses initially, it is unclear whether they can induce durable immune memory against tumour cells. Cancer vaccines need to generate long-lasting immunity to prevent recurrence, especially for tumours with high metastatic potential. Researchers must explore whether OMV-based vaccines can elicit long-term memory T cells that can persist and provide ongoing protection.

Ethical and Ecological Considerations: The use of genetically modified bacteria to produce OMVs, particularly on a large scale, raises ethical and ecological concerns. The risks associated with unintentional release or mismanagement of genetically engineered bacteria must be addressed through strict safety protocols. Additionally, there could be unforeseen consequences of widespread use, such as antibiotic resistance or unintended impacts on the microbiome.

Critical Thinking and Future Directions: Given the promise and challenges of engineered OMVs, several critical questions emerge for future research and development:

How can OMVs be optimised to deliver antigens more precisely and consistently across different patient populations and cancer types?

What strategies can be employed to modulate the immune response to OMVs?

Can engineered OMVs overcome the immune-suppressive effects of the tumour microenvironment and sustain long-term immune memory against tumours?

What will be the cost and practicality of scaling OMV production for clinical use, and how can manufacturing be standardised across diverse vaccine formulations?

How can regulatory frameworks adapt to accommodate the novel nature of OMV-based therapies, balancing innovation with safety?

In conclusion, while engineered OMVs represent an exciting advancement in cancer vaccines, the field requires careful navigation of technical, immunological, and regulatory challenges. Through critical research and innovation, OMV-based vaccines could eventually offer a powerful tool in the fight against cancer, but ongoing work is needed to address the complexities of safety, efficacy, and real-world application.

## Figures and Tables

**Table 1:** Engineered outer membrane vesicles for tumour imaging.

Cancer Types	Material Properties	Bioengineering Method	References	Preclinical Model and Evaluation
Murine breast cancer	Biopolymer-melanin	*E. coli* K12 expressing a tyrosinase transgene to produce OMVs encapsulating biopolymer-melanin OMV^Mel^	Gujrati V et al., 2019 [77]	Bioengineered OMV^Mel^ was injected into a mouse breast cancer model for tumour imaging; may enable real-time three-dimensional theranostic applications of OMV^Mel^ in simultaneous optoacoustic imaging and cancer therapeutics
Murine melanoma	Hollow polydopamine	OMV–CC hybrid membrane coated onto hollow polydopamine (HPDA) NPs	Wang et al., 2020 [78]	Hollow polydopamine (HPDA)-coated engineered OMVs (OMV-CC) were utilised to treat a murine melanoma model; provides adaptability for various synergistic therapeutic and imaging applications by incorporating payload with application-specific functions
Murine breast cancer	Cationic dyes	OMVs and cationic dyes mixed by shaking in an incubator	Liu et al., 2023 [79]	An OMV-loaded cationic dye was injected into a mouse breast cancer model for tumour imaging detection; function as biocompatible nanocarriers to efficiently deliver cationic molecules for tumour theranostic applications
Colon cancer	Iodine-131	High tyrosine-rich protein statherin (STATH) was integrated onto the surfaces of attenuated Salmonella typhimurium-derived OMVs	Shen et al., 2024 [37]	An engineered OMV-based delivery system (131I-STATH-OMV-PEG) was utilised for radioimmunotherapy in an orthotopic colon cancer model; amplifies the therapeutic effect of RIT and offers a promising approach for tumour radioimmunotherapy

**Table 2:** Modification of outer membrane vesicles for improved special targeting.

Cancer Types	Target Protein	Bioengineering Method	References	Preclinical Evaluation
Murine breast cancer	EGFR	A fusion protein of ClyA-EGFR-scFv expression on OMVs	Rezaei et al., 2023 [89]	Anti-EGFR-engineered outer membrane vesicles (OMVs) were utilised to treat a mouse model of triple-negative breast cancer; in vitro results demonstrated that scFv-OMVs effectively targeted EGFR on the surfaces of various high EGFR-expressing cancer cells, and in vivo studies support the potential application of OMVs as immunotherapy agents
Murine breast cancer	EGFR	Fusion of the affi-EGFR-GALA structure to the C-terminal of ClyA as an anchor protein	Sepahdar et al., 2021 [90]	Engineered OMVs derived from *E. coli* and modified with an EGFR-GALA peptide fusion protein was used to target EGFR-overexpressing breast cancer cell lines in a preclinical model; engineered OMVs targeting EGFR in triple-negative breast cancer cells provide a potential avenue for specific antitumour therapy with low toxicity
Murine melanoma/Colon cancer	PD-L1	A fusion protein of ClyA-PD-L1 on OMVs	Li et al. and Cui et al., 2020 [75,92]	Engineered OMV-PD-1 vesicles can bind to PD-L1 on the surfaces of tumour cells, providing a targeted treatment approach for colon cancer and melanoma in mouse models; Engineered OMV-PD-1 can bind to PD-L1 on the tumour cell surface and facilitate its internalisation and reduction, thereby protecting T cells from the PD-1/PD-L1 immune inhibitory axis
Murine breast cancer	Nucleolin	Integration of an AS1411 aptamer to OMV surfaces via hydrophobic interactions	Chen et al. [94]	DNA aptamer assembly on outer membrane vesicles (Apt-OMV) enhances tumour targeting, providing an effective treatment approach for triple-negative breast cancer in mouse models; Apt-OMVs took advantage of the spherical nucleic acid structure to shield OMVs against nonspecific immune recognition and evade immunogenicity
Thyroid cancer	Mammalian cell membrane	Utilising a Poly(L-arginine) cell penetrating peptide (CPP) to enhance OMV affinity	Suyang Wang et al., 2024 [96]	Engineered OMVs (SOMV-9RE7) effectively activated adaptive immunity for the treatment of TC-1 tumour-bearing mouse model; SOMV-9RE7 exhibited promising antitumour effects by generating systemic E7-specific CD8^+^ T cells and recruiting them to the tumour microenvironment
Murine breast cancer/colon cancer	ECM	A fusion peptide, cytolysin A (ClyA)-hyaluronidase (Hy) was expressed on outer membrane vesicles	Shindu C Thomas et al., 2021 [98,99]	Engineered OMVs (ΔECHy) incorporating a cytolysin A (ClyA)-hyaluronidase (Hy) fusion protein was utilised to treat mouse models of breast and colon cancer; ΔECHy combined with immune checkpoint antibodies and tyrosine kinase inhibitors remodelled the tumour stroma, resulting in the improvement of immunotherapy outcomes and enhancing the efficacy of biological signalling inhibitors
Murine bladder tumour	RMF orientation	OMVs conjugated with TiO2-coated Fe3O4 NPs	Li et al., 2023 [102]	Motile microrobots loaded with magnetic nanocores and engineered OMVs were utilised to treat melanoma in mouse models; actively delivered cargo to malignant glioma in vivo. *E. coli* membrane camouflaging enhances the efficiency of phagocytosis and also prevents drug leakage inside neutrophils
Murine breast cancer	CD206	Biomembrane hydrogel based on mannose-decorated OMVs	Xinxin Nie et al., 2023 [18]	A mannose-decorated hybrid biomembrane (MHCM) modified with oxidised sodium alginate (OSA) was designed as a gelator (O-MHCM) and used to treat a mouse model of breast cancer; the MHCM enables active targeting to dendritic cells (DCs) and effective DC maturation, sufficient antigen availability, and strong and sustainable T lymphocyte-mediated immunity

## Supplementary Materials

The following are available online at https://www.mdpi.com/article/10.3390/pharmaceutics17010058/s1, Table S1: OMV surface modified protein and amino acid sequence.
