# Supplementary material for: Innovative Strategies in Oncology: Bacterial Membrane Vesicle-Based Drug Delivery Systems for Cancer Diagnosis and Therapy
Source: Pharmaceutics. 2025 Jan 3;17(1):58. doi: 10.3390/pharmaceutics17010058 (PMC11768367; doi:10.3390/pharmaceutics17010058)
Supplement: Supplementary file 1 [file pharmaceutics-17-00058-s001.zip › pharmaceutics-3345188-supplementary.pdf]

Materials and methods

Molecular Docking

The crystal structure was retrieved from the protein databank (PDB) (MHC class I heavy chain complex with BETA-2 microglobulin and chicken ovalbumin, PDB: 1VAC; Cytolysin A, PDB: 2WD; DSFV MR1 and EGFRVIII peptide antigen complex, PDB: 1I8I; TLR4 and MD-2 complex, PDB: 3FXI; CD47 and SIRPα complex, PDB: 7YGG; CD47 antibody Fab in complex with CD47, PDB: 7XJF; Human PD1 and PD-L1 complex, PDB: 4ZQK; BFGF, PDB: 2FgF; EGFR, PDB: 7U98; EGFR nanobody, PDB: 7OM5). The amino acid sequence of HPV16E7 has been reported and used as a reference [66]. The 3D structure of HPV16E7 was predicted via submission of the amino acid sequence to the AlphaFold Server, and the top 1 prediction structure was downloaded. 3D structural analysis and molecular docking studies were performed by using MOE, and the top 1 molecular docking result was used for analysis.

Table S1. OMV surface modified protein and amino acid sequence.

Protein	Amino acids
EGFR	Asp 807, Val 980, Arg 803, and Lys 806
Anti-EGFR nanobodies	Gln 39, Gln 114, Val 107, Tyr 109, and Asn 106
PD1	Phe 19, Asp 26, Gln 66, Ala 121, Asp 122, Tyr 123, Lys 124, Arg 125
PD-L1	Asn 66, Tyr 68, Gln 75, Asp 77, Lys 78, Ala 132, and Glu 136
Ovalbumin	Ser 1, Ile 2, Ile 3, Asn 4, Phe 5, Glu 6, Lys 7, and Leu 8
EGFRvIII peptide antigen	Glu 499, Glu 500, Lys 501, Lys 502, Gly 503, Asn 504, Tyr 505, Val 506, Val 507, Thr 508, Asp 509, His 510
